# The mediating role of depression between adverse childhood experiences and violent discipline among Chinese parents of preschool children

**DOI:** 10.7717/peerj.21130

**Published:** 2026-04-28

**Authors:** Fei Yang, Li Yin, Xinxuan Li, Mo Zhou, Ziyuan Fu, Zhaoxue Meng, Hong Zhou

**Affiliations:** 1 Department of Maternal and Child Health, School of Public Health, Peking University, Beijing, China; 2 Weifang Mother and Child Health Care Institution, Shandong, China; 3 Tongzhou District Mother and Child Health Care Institution, Beijing, China; 4 National Health Commission Key Laboratory of Reproductive Health, Peking University School of Public Health, Beijing, China; 5 Peking University Health Science Center—Weifang Joint Research Center for Maternal and Child Health, Beijing, China

**Keywords:** Adverse childhood experiences, Violent discipline, Depression, Parents of preschoolers

## Abstract

**Background:**

Violent discipline toward children remains common worldwide and has been linked to parents’ adverse childhood experiences (ACEs), suggesting intergenerational transmission. Parental depression may play an important role in this pathway. This study examined whether parental depression mediates the association between ACEs and violent discipline among Chinese parents of preschool children.

**Methods:**

This cross-sectional study included 1,650 parents of preschool children in Tongzhou District, Beijing. Parents reported their ACEs, depressive symptoms, and use of violent discipline. Logistic regression and generalized structural equation modeling were employed to explore the relationships among ACEs, violent discipline, and depression.

**Results:**

Overall, 71.9% reported using violent discipline, and 38.4% reported at least one type of ACE. Higher ACE exposure was associated with greater use of violent discipline. Parental depression was positively associated with both ACEs and violent discipline and partially mediated the association between ACEs and violent discipline, including psychological aggression, physical punishment, and severe physical punishment.

**Conclusions:**

Parental ACEs were associated with violent discipline among Chinese parents of preschool children, and depression partially explained this association. These findings highlight the importance of addressing parental depression in interventions aimed at reducing violent discipline and interrupting intergenerational cycles of adversity.

## Introduction

Violent discipline within the family is the most prevalent form of violence against children globally (UNICEF, 2024) and includes two main dimensions: psychological aggression and physical punishment (Bhatia et al., 2020). Globally, nearly three-quarters of children aged 2–4 years are frequently subjected to violent discipline by parents or other caregivers (UNICEF, 2017), and around six in ten children aged 2–14 years regularly experience physical punishment (UNICEF, 2014). Violent discipline has been associated with a range of adverse outcomes, including developmental delays and psychiatric disorders that harm children’s physical and psychological well-being (Islam, 2024; McLaughlin, Sheridan & Lambert, 2014; Schlensog-Schuster et al., 2024), as well as an increased risk of drug abuse, crime, and other social problems later in life (Bunch, Iratzoqui & Watts, 2018; Hughes et al., 2017; Zhu et al., 2025).

Adverse childhood experiences (ACEs) refer to potentially traumatic events occurring before age 18, including abuse, neglect, and household dysfunction (Felitti et al., 1998). These experiences are associated with long-term effects on adult behavioral patterns, particularly parenting practices (Rowell & Neal-Barnett, 2021; Wattanatchariya, Narkpongphun & Kawilapat, 2024). A growing body of evidence indicates an association between parents’ ACEs and their subsequent use of violent discipline in adulthood (Afifi et al., 2022; Madigan et al., 2019; Scheid, Miller-Graff & Guzmán, 2021; Shin et al., 2023). This pathway from parental ACEs to the use of violent discipline suggests an intergenerational transmission of adversity within families. Further research is needed to elucidate the mechanisms underlying this transmission and to inform the development of effective interventions (Schelbe & Geiger, 2017).

Emerging evidence suggests that mental health among parents with ACEs represents a modifiable target for interventions addressing intergenerational risk (Assink et al., 2018; Dube, 2025; Dube & Rishi, 2017; Kirkbride et al., 2024; Narayan, Lieberman & Masten, 2021). In particular, parental depression, as one of the most prevalent mental disorders, represents a potential link between parental adversity and child outcomes. On one hand, previous studies suggest that children who experience early trauma are at increased risk of developing depression in adulthood (Gardner, Thomas & Erskine, 2019; Guo et al., 2023; Merrick et al., 2017; Shi et al., 2025; Wang et al., 2023; Yu et al., 2024; Zhang et al., 2023). On the other hand, research indicates that parents with depression are more likely to use violent behaviors toward children. For example, research has shown that mothers with depression report higher rates of both psychological and physical aggression against their children (Marçal, 2021; Wolford, Cooper & McWey, 2019). Similarly, fathers with depression are more likely to engage in negative parenting behaviors (Wilson & Durbin, 2010) and abuse or physically punish their children (Kelley et al., 2015; Lee et al., 2011).

Although prior evidence has suggested the potential mediating role of depression in the intergenerational cycle of ACEs, existing studies have primarily focused on parent populations from high-income countries (Choi et al., 2019; Dixon, Browne & Hamilton-Giachritsis, 2005; Mapp, 2006; Morelli et al., 2021; Schuetze & Eiden, 2005). Evidence from developing countries with diverse parenting cultural contexts remains limited, and data from mainland China are relatively scarce. China, characterized by rapid socioeconomic transition, has a traditional parenting culture expressed by the sayings ‘sparing the rod spoils the child’ and ‘strict discipline is a symbol of love’ (Liu & Wang, 2015; Zhu et al., 2024). The use of strict discipline has persisted across generations as an ingrained cultural practice in Chinese households. Research evidence indicates that the prevalence of ACEs among the Chinese population is relatively high. For example, an analysis of data from the China Health and Retirement Longitudinal Study (CHARLS) found that 86.4% of adult Chinese residents reported exposure to at least one ACE (Xie, Fang & Zeng, 2023). Concurrently, rates of violent discipline remain notably high in mainland China. A large-scale national survey revealed that 53.7% of mothers and 48.3% of fathers reported using corporal punishment, while 80.3% of mothers and 74.9% of fathers acknowledged using psychological aggression toward their children (Wang & Liu, 2014). Furthermore, studies have confirmed a direct association between parental ACEs and violent discipline (Shen & Wu, 2024; Zhu et al., 2024), reflecting patterns of intergenerational transmission within Chinese families. Understanding how to break this intergenerational cycle has emerged as a critical focus in recent studies targeting the Chinese population. Promoting the comprehensive and healthy development of children is central to these efforts.

Therefore, this study examines the mediating role of depression between ACEs and violent discipline among Chinese parents of preschool children. Rather than focusing solely on cross-cultural validation of a theoretical model, this study aims to identify modifiable risk factors within China’s socio-cultural context. The findings may provide empirical evidence to inform the development of targeted interventions addressing the intergenerational association between ACEs and violent discipline.

## Materials and Methods

### Study design and participants

This cross-sectional survey was conducted in kindergartens in the Tongzhou District, Beijing, in collaboration with the Tongzhou District Mother and Child Health Care Institution. We conducted a self-administered, anonymous survey among the caregivers involved in early childhood development. The inclusion criteria were: (1) the caregiver was the child’s father or mother, and (2) the child was a preschooler aged 3–6 years. Prior to survey administration, researchers trained field staff by outlining the purpose and significance of the study and emphasizing data confidentiality. This study was approved by the Ethics Committee of the Beijing Tongzhou District Mother and Child Health Care Institution (2021-TZFY-041-01), and all participants provided written informed consent before completing the questionnaire.

Based on reported prevalence rates of violent discipline among Chinese preschoolers ranging from 44.9% to 72.6% (Cui et al., 2016; Huang et al., 2020; Qin, Du & Chen, 2023; Wei et al., 2016), we used the median prevalence (60%) for sample size estimation. Applying the cross-sectional study formula N = [Z^2^ × P(1 − P)]/e^2^ (Z = 1.96, e = 0.03, *P* = 0.60), the minimum required sample size was 1,024. To account for the needs of mediation analysis (which typically requires a sample size 30–50% larger than the minimum), as well as subgroup analysis and an anticipated 15% non-response rate, we aimed to recruit between 1,500 and 1,800 participants.

Between January and April 2022, 1,798 questionnaires were collected. During data cleaning, questionnaires were excluded if they: (1) contained duplicate responses or incomplete information; (2) were completed by non-parent caregivers; or (3) referred to a child outside the 3–6-year age range. The final sample included 1,650 valid questionnaires, yielding a 91.8% response validity rate. This sample size was sufficient to ensure regional representativeness and to meet the requirements of complex mediation modeling while maintaining statistical reliability.

### Measures

This study used the Behavioral Risk Factor Surveillance System (BRFSS) ACE questionnaire (2009–2021) to measure ACEs experienced during the first 18 years of the parents’ lives (Centers for Disease Control and Prevention, 2023). The BRFSS ACE questionnaire (2009–2021) covers 11 items across eight modules and collects information on experiences of child abuse, neglect, and household challenges. In U.S. population-based studies, the BRFSS ACE questionnaire has demonstrated acceptable to strong internal consistency, with Cronbach’s α ranging from 0.61 to 0.80 across different scales and an overall Cronbach’s α of 0.78 for the full questionnaire (Ford et al., 2014). The questionnaire includes eight types of ACEs: emotional abuse (one item), physical abuse (one item), sexual abuse (three items), mental illness in the household (one item), incarcerated household member (one item), parental separation or divorce (one item), intimate partner violence (one item), and substance abuse in the household (two items). Each type of ACE was coded as “0” if never experienced, and “1” if experienced. The total ACE score was calculated by summing the scores across all items for each participant (Peterson et al., 2023; Swedo et al., 2023). Additionally, to assess cumulative exposure to ACEs, each participant’s total ACE score was trichotomized into 0 types of ACEs, 1–2 types of ACEs, and 3 or more types of ACEs (Gupta, 2022). The Cronbach’s α for the questionnaire in this study sample was 0.732.

Depression was evaluated using the Self-Rating Depression Scale (SDS) developed by Zung, Richards & Short (1965), which assesses self-reported depressive symptoms over the past 2 weeks. Previous studies have demonstrated that the SDS has good reliability and validity among the Chinese population (Peng et al., 2013; Wang et al., 2014; Zhang et al., 2012). The SDS consists of 20 items rated on a 4-point Likert scale. Ten items are positively worded (almost never = 4, sometimes = 3, often = 2, always = 1), and the remaining ten items are reverse-worded (almost never = 1, sometimes = 2, often = 3, always = 4). The raw total score ranges from 20 to 80. Standardized scores were calculated by multiplying the raw total score by 1.25 and rounding to the nearest integer. Higher scores indicate greater depression severity. The Cronbach’s α for the scale in this study sample was 0.838.

Violent discipline was a dichotomous variable (yes/no) covering both psychological aggression and physical punishment, based on the global Multiple Indicator Cluster Surveys (MICS) program (UNICEF, 2020). Psychological aggression was defined as the use of at least one of the following two behaviors: (1) shouted, yelled, or screamed; (2) called dumb, lazy, or another name. Physical punishment was defined as the use of at least one of the following six behaviors: (1) shook him/her; (2) spanked, hit, or slapped on the bottom with a bare hand; (3) hit with a belt, hairbrush, stick, or other hard object; (4) hit or slapped on the face, head, or ears; (5) hit or slapped on the hand, arm, or leg; (6) beat up, hit over and over as hard as one could. If the answer to item (4) or (6) was yes, the behavior was further defined as severe physical punishment. Reporting any form of psychological aggression or physical punishment was classified as experiencing violent discipline. The Cronbach’s α for all eight items in this study sample was 0.739.

**Figure 1:**
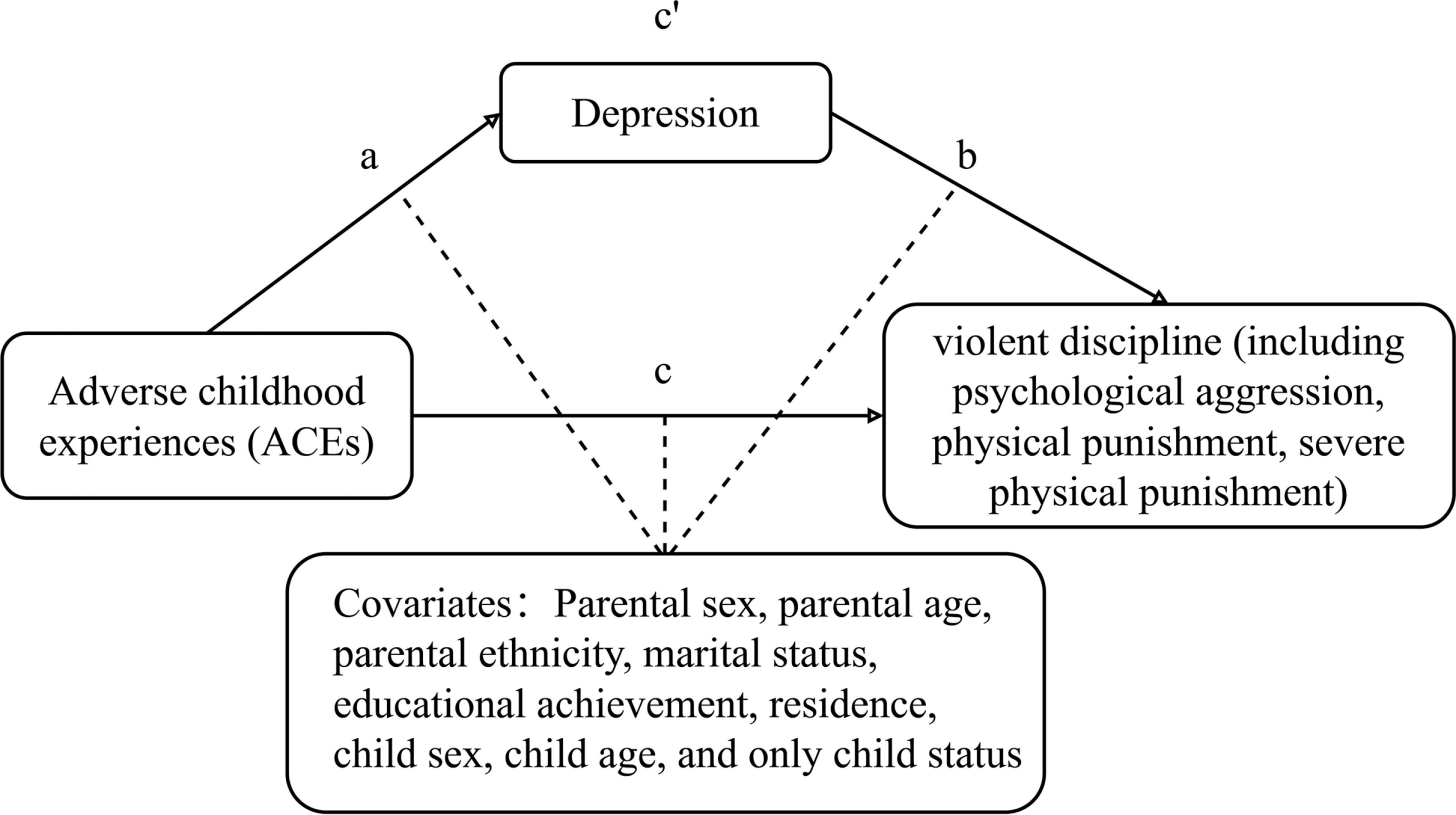
Conceptual diagram of the mediating effect of depression on the relationship between ACEs and violent discipline. a, the path between the independent variable (ACEs) and the mediator (depression) after controlling for covariates; b, the path between the mediator (depression) and the dependent variable (including violent discipline, psychological aggression, physical punishment, and severe physical punishment) after controlling for the independent variable (ACEs) and covariate; c, the path between the independent variable (ACEs) and the dependent variable (including violent discipline, psychological aggression, physical punishment, and severe physical punishment) after controlling for covariates, without the mediator (depression); c′, the path between the independent variable (ACEs) and the dependent variable (including violent discipline, psychological aggression, physical punishment, and severe physical punishment) after controlling for covariates with the mediator (depression) included.

We controlled for both parental and child covariates. Parental characteristics included sex (male/female), age (<30, 30–34, 35–39, ≥40 years), ethnicity (Han/minority), marital status (married/other), educational achievement (middle school or below, high school or vocational school, college or university, master’s or above), and residence (urban/rural). Child characteristics included sex (male/female), age (3, 4, and 5–6 years), and only-child status (yes/no) (Fig. 1).

### Statistical analysis

First, descriptive analyses were performed to describe the characteristics of parents and children, as well as ACEs, depression, and violent discipline. Subsequently, univariable analyses were conducted to examine between-group differences in the prevalence of violent discipline. Then, logistic regression analyses were conducted to explore the association between ACEs and violent discipline. Four models were established for each type of violent discipline, and all were adjusted for relevant covariates. Odds ratios (ORs) and 95% confidence intervals (CIs) were calculated. Finally, Generalized Structural Equation Modeling (GSEM) was used to analyze the mediating effect of depression on the association between ACEs and violent discipline after adjusting for covariates. GSEM is an extended application of Structural Equation Modeling (SEM) that can handle various forms of data, including continuous, binary, count, categorical, ordinal, fractional, and survival-time data (StataCorp LLC, 2024). It has also been applied to the analysis of mediating effects (Muthén & Asparouhov, 2014). The mediation analysis yielded estimates of the mediation effect (ME), direct effect (DE), total effect (TE), and proportion mediated (PM), along with 95% confidence intervals. All statistical analyses were performed using *Stata 17.0* and *SPSS 25.0*. *P* < 0.05 was considered statistically significant.

## Results

Descriptive statistics showed that among the 1,650 parents, the majority were mothers, accounting for 84.7%, while fathers made up 15.3%. Most of the parents were aged between 30 and 40 years (73.5%), of Han ethnicity (93.9%), and married (98.5%). Regarding educational achievement, participants generally reported high education levels: 75.7% of parents held a college or university degree, 10.6% had completed high school or vocational school, 9.5% had a master’s degree or higher, and only 4.2% had a middle school education or below. Moreover, the majority of families resided in urban areas (87.8%), with a smaller proportion living in rural areas (12.2%). Regarding the characteristics of the children, boys and girls were almost equally represented (50.5% and 49.5%, respectively), with 54.3% being only children.

Among the 1,650 parents, 71.9% reported using violent discipline with their children: 63.2% had used psychological aggression, 48.6% had physically punished their children, and 4.6% had inflicted severe physical punishment. The prevalence of violent discipline varied by the child’s age (χ^2^ = 6.844, *P* = 0.033). The prevalence of psychological aggression varied by both the parent’s age (χ^2^ = 11.165, *P* = 0.011) and the child’s age (χ^2^ = 8.914, *P* = 0.012). The prevalence of physical punishment varied by the parent’s educational achievement (χ^2^ = 9.486, *P* = 0.023) and the child’s sex (χ^2^ = 7.402, *P* = 0.007). The prevalence of severe physical punishment varied by the parent’s educational achievement (χ^2^ = 8.906, *P* = 0.031) and the child’s sex (χ^2^ = 8.804, *P* = 0.003) (Table 1).

**Table 1:** Prevalence of violent discipline by parent and child characteristics (*n* = 1,650).

Characteristic	Total	Violent discipline	Psychological aggression	Physical punishment	Severe physical punishment
	*n* (%)	*n* (%)	*n* (%)	*n* (%)	*n* (%)
**Total**	1,650 (100.0)	1,187 (71.9)	1,042 (63.2)	801 (48.6)	76 (4.6)
**Parental sex**					
Male	253 (15.3)	184 (72.7)	159 (62.9)	131 (51.8)	15 (5.9)
Female	1,397 (84.7)	1,003 (71.8)	883 (63.2)	670 (48.0)	61 (4.4)
$\chi^2$		0.092	0.012	1.251	1.190
*P*-value^①^		0.762	0.913	0.263	0.275
**Parental age**					
<30	159 (9.6)	117 (73.6)	104 (65.4)	81 (50.9)	13 (8.2)
30–34	673 (40.8)	493 (73.3)	441 (65.5)	333 (49.5)	32 (4.8)
35–39	539 (32.7)	392 (72.7)	345 (64.0)	256 (47.5)	19 (3.5)
≥40	279 (16.9)	185 (66.3)	152 (54.5)	131 (47.0)	12 (4.3)
$\chi^2$		5.338	11.165	1.122	6.139
*P*-value^①^		0.149	0.011	0.772	0.105
**Parental ethnicity**					
Han	1,550 (93.9)	1,111 (71.7)	982 (63.4)	748 (48.3)	72 (4.7)
Minority	100 (6.1)	76 (76.0)	60 (60.0)	53 (53.0)	4 (4.0)
$\chi^2$		0.870	0.454	0.846	0.003
*P*-value^①^		0.351	0.500	0.358	0.958
**Marital status**					
Married	1,625 (98.5)	1,168 (71.9)	1,025 (63.1)	786 (48.4)	74 (4.6)
Other	25 (1.5)	19 (76.0)	17 (68.0)	15 (60.0)	2 (8.0)
$\chi^2$		0.207	0.256	1.333	0.112
*P*-value^①^		0.649	0.613	0.248	0.738
**Educational achievement**					
Middle school or below	70 (4.2)	46 (65.7)	35 (50.0)	37 (52.9)	5 (7.1)
High school or vocational school	174 (10.6)	131 (75.3)	114 (65.5)	96 (55.2)	15 (8.6)
College or university	1,249 (75.7)	902 (72.2)	799 (64.0)	607 (48.6)	51 (4.1)
Master’s or above	157 (9.5)	108 (68.8)	94 (59.9)	61 (38.9)	5 (3.2)
$\chi^2$		3.129	6.707	9.486	8.906
*P*-value^①^		0.372	0.082	0.023	0.031
**Residence**					
Urban	1,448 (87.8)	1,038 (71.7)	914 (63.1)	696 (48.1)	67 (4.6)
Rural	202 (12.2)	149 (73.8)	128 (63.4)	105 (52.0)	9 (4.5)
$\chi^2$		0.379	0.005	1.087	0.012
*P*-value^①^		0.538	0.946	0.297	0.913
**Child’s sex**					
Male	833 (50.5)	609 (73.1)	528 (63.4)	432 (51.9)	51 (6.1)
Female	817 (49.5)	578 (70.8)	514 (62.9)	369 (45.2)	25 (3.1)
$\chi^2$		1.141	0.040	7.402	8.804
*P*-value^①^		0.286	0.842	0.007	0.003
**Child’s age**					
3	415 (25.2)	278 (67.0)	237 (57.1)	194 (46.8)	16 (3.9)
4	600 (36.4)	439 (73.2)	395 (65.8)	307 (51.2)	36 (6.0)
5–6	635 (38.5)	470 (74.0)	410 (64.6)	300 (47.2)	24 (3.8)
$\chi^2$		6.844	8.914	2.618	4.173
*P*-value^①^		0.033	0.012	0.270	0.124
**Only child**					
Yes	896 (54.3)	645 (72.0)	571 (63.7)	418 (46.7)	38 (4.2)
No	754 (45.7)	542 (71.9)	471 (62.5)	383 (50.8)	38 (5.0)
$\chi^2$		0.002	0.280	2.815	0.595
*P*-value^①^		0.963	0.597	0.093	0.441

**Note:**

^①^ Pearson’s Chi-squared test or Yates’s correction for continuity.

Among the 1,650 parents, 61.6% reported no ACEs, 25.2% reported 1–2 types of ACEs, and 13.2% reported ≥3 types of ACEs. The prevalence of violent discipline (χ^2^ = 45.142, *P* < 0.001), psychological aggression (χ^2^ = 74.514, *P* < 0.001), physical punishment (χ^2^ = 18.070, *P* < 0.001) and severe physical punishment (χ^2^ = 42.011, *P* < 0.001) increased with parental ACE count. The median standardized depression score for the parents was 43. The depression scores of the parents who reported violent discipline (Z = 3.474, *P* < 0.001), psychological aggression (Z = 3.681, *P* < 0.001), physical punishment (Z = 4.984, *P* < 0.001) and severe physical punishment (Z = 7.207, *P* < 0.001) were higher than those of parents who had never employed such behaviors, suggesting that parents who reported violent discipline had significantly higher depression scores (Table 2).

**Table 2:** Incidence of violent discipline by parental ACEs and depression (*n* = 1,650).

Characteristic	Total	Violent discipline	Psychological aggression	Physical punishment	Severe physical punishment
	*n* (%)	*n* (%)	*n* (%)	*n* (%)	*n* (%)
**ACEs**					
0	1,017 (61.6)	672 (66.1)	560 (55.1)	452 (44.4)	25 (2.5)
1–2	415 (25.2)	338 (81.5)	316 (76.1)	226 (54.5)	24 (5.8)
≥3	218 (13.2)	177 (81.2)	166 (76.2)	123 (56.4)	27 (12.4)
$\chi^2$		45.142	74.514	18.070	42.011
*P*-value^①^		<0.001	<0.001	<0.001	<0.001
**Depression** ^②^	43 (35,50)				
**(Have employed)** ^②^		43 (35,50)	44 (36,50)	44 (36,51)	54 (44.5,61)
**(Never employ)** ^②^		40 (34,49)	40.5 (34,49)	41 (34,48)	41 (35,49)
Z		3.474	3.681	4.984	7.207
*P*-value^①^		<0.001	<0.001	<0.001	<0.001

**Notes:**

^①^ Pearson’s Chi-squared test or Wilcoxon rank-sum test, as appropriate.

^②^ Quartile.

According to the results of logistic regression, after controlling for covariates, parental ACEs were associated with increased odds of violent discipline, psychological aggression, physical punishment, and severe physical punishment. Compared to parents without ACEs, parents with 1–2 types of ACEs were more likely to engage in violent discipline (OR 2.18, 95% CI [1.64–2.89]), psychological aggression (OR 2.51, 95% CI [1.93–3.26]), physical punishment (OR 1.47, 95% CI [1.16–1.85]), and severe physical punishment (OR 2.35, 95% CI [1.29–4.25]). Similarly, parents with ≥3 types of ACEs exhibited the same pattern (violent discipline: OR 2.08, 95% CI [1.44–3.02]; psychological aggression: OR 2.40, 95% CI [1.70–3.38]; physical punishment: OR 1.50, 95% CI [1.11–2.04]; severe physical punishment: OR 4.31, 95% CI [2.34–7.94]). Furthermore, each 1-point increase in depression score was associated with higher odds of violent discipline (OR 1.01, 95% CI [1.00–1.02]), psychological aggression (OR 1.01, 95% CI [1.00–1.02]), physical punishment (OR 1.02, 95% CI [1.01–1.03]), and severe physical punishment (OR 1.08, 95% CI [1.06–1.11]).

With respect to parental and child characteristics, compared with parents aged 40 years or older, parents aged 30–34 years had higher odds of using violent discipline (OR 1.42, 95% CI [1.03–1.96]). Parents younger than 30, 30–34, and 35–39 years showed ORs of 1.64 (95% CI [1.06–2.54]), 1.61 (95% CI [1.18–2.18]) and 1.46 (95% CI [1.07–1.98]) for psychological aggression, respectively. Compared with parents holding a master’s degree or above, parents with a high school or vocational school and those with a college or university degree had ORs of 1.77 (95% CI [1.11–2.81]) and 1.49 (95% CI [1.05–2.11]) for physical punishment, respectively. Compared with boys, girls had lower odds of being physically punished (OR 0.72, 95% CI [0.59–0.88]) and severely physically punished (OR 0.36, 95% CI [0.21–0.61]). Relative to 3-year-old children, 4-year-olds and 5–6-year-olds had ORs of 1.33 (95% CI [1.00–1.76]) and 1.42 (95% CI [1.07–1.88]) for experiencing violent discipline, respectively. Additionally, children aged 4 years and those aged 5–6 years had ORs of 1.46 (95% CI [1.11–1.90]) and 1.42 (95% CI [1.09–1.85]) for experiencing psychological aggression, respectively. Compared with only children, non-only children had an OR of 1.25 (95% CI [1.02–1.54]) for being physically punished (Table 3).

**Table 3:** Association between ACEs of parents and violent discipline (*n* = 1,650).

Characteristic	Violent discipline	Psychological aggression	Physical punishment	Severe physical punishment
	OR (95% CI)^①②^	OR (95% CI)^①②^	OR (95% CI)^①②^	OR (95% CI)^①②^
**ACEs**				
(0)	1.00	1.00	1.00	1.00
1–2	2.18*** [1.64–2.89]	2.51*** [1.93–3.26]	1.47** [1.16–1.85]	2.35** [1.29–4.25]
≥3	2.08*** [1.44–3.02]	2.40*** [1.70–3.38]	1.50** [1.11–2.04]	4.31*** [2.34–7.94]
**Depression**	1.01* [1.00–1.02]	1.01* [1.00–1.02]	1.02*** [1.01–1.03]	1.08*** [1.06–1.11]
**Parental sex**				
(Male)	1.00	1.00	1.00	1.00
Female	0.96 [0.70–1.31]	1.02 [0.77,1.37]	0.86 [0.65–1.14]	0.81 [0.43–1.53]
**Parental age**				
(≥40)	1.00	1.00	1.00	1.00
<30	1.43 [0.90–2.27]	1.64* [1.06–2.54]	1.10 [0.73–1.67]	1.70 [0.68–4.25]
≥30 & <35	1.42* [1.03–1.96]	1.61** [1.18–2.18]	1.17 [0.87–1.58]	1.21 [0.58–2.54]
≥35 & <40	1.34 [0.97–1.86]	1.46* [1.07–1.98]	1.04 [0.77–1.41]	0.74 [0.34–1.62]
**Parental ethnicity**				
(Han)	1.00	1.00	1.00	1.00
Minority	1.36 [0.84–2.20]	0.90 [0.59–1.39]	1.38 [0.91–2.09]	1.29 [0.43–3.80]
**Marital status**				
(Married)	1.00	1.00	1.00	1.00
Otherwise	1.06 [0.41–2.74]	1.12 [0.46–2.71]	1.54 [0.67–3.52]	1.58 [0.33–7.62]
**Educational achievement**				
(Master’s or above)	1.00	1.00	1.00	1.00
Middle school or below	0.84 [0.44–1.60]	0.64 [0.35–1.18]	1.59 [0.87–2.91]	1.52 [0.37–6.16]
High school or vocational school	1.27 [0.76–2.12]	1.15 [0.71–1.87]	1.77* [1.11–2.81]	1.63 [0.53–5.02]
College or university	1.17 [0.80–1.70]	1.16 [0.81–1.66]	1.49* [1.05–2.11]	1.03 [0.39–2.73]
**Residence**				
(Urban)	1.00	1.00	1.00	1.00
Rural	1.03 [0.72–1.48]	0.98 [0.71–1.37]	0.99 [0.72–1.35]	0.65 [0.30–1.43]
**Child sex**				
(Male)	1.00	1.00	1.00	1.00
Female	0.85 [0.68–1.06]	0.94 [0.76–1.15]	0.72** [0.59–0.88]	0.36*** [0.21–0.61]
**Child age**				
(3 years old)	1.00	1.00	1.00	1.00
4 years old	1.33* [1.00–1.76]	1.46** [1.11–1.90]	1.16 [0.90–1.50]	1.63 [0.86–3.09]
5–6 years old	1.42* [1.07–1.88]	1.42* [1.09–1.85]	0.99 [0.77–1.28]	1.09 [0.54–2.18]
**Only child**				
(Yes)	1.00	1.00	1.00	1.00
No	1.09 [0.86–1.38]	1.07 [0.86–1.33]	1.25* [1.01–1.54]	1.46 [0.88–2.44]

**Notes:**

^①^*P < 0.05, **P < 0.01, ***P < 0.001.

^②^ OR, odds ratio; CI, confidence interval.

The mediation analysis indicated that parental depression partially mediated the association between ACEs and violent discipline (95% CI [0.000–0.012], *P* < 0.05), psychological aggression (95% CI [0.000–0.012], *P* < 0.05), physical punishment (95% CI [0.005–0.019], *P* < 0.001), and severe physical punishment (95% CI [0.005–0.012], *P* < 0.001). The proportions mediated were 6.55%, 4.81%, 16.76%, and 18.81%, respectively (Table 4).

**Table 4:** The mediating effect of depression on the relationship between ACEs and violent discipline (*n* = 1,650).

Variable	Coef. (95% CI)/proportion^①②^	*P*-value
**Violent discipline**		
a	2.53 [1.85–3.21]	<0.001
b^③^	1.02 [1.01–1.03]	0.001
Causal mediation effect (c′)	0.006 [0.000–0.012]	0.034
Direct effect (c)	0.088 [0.058–0.118]	<0.001
Total effect	0.094 [0.064–0.124]	<0.001
Proportion of mediation	6.55%	
**Psychological aggression**		
a	2.53 [1.85–3.21]	<0.001
b^③^	1.02 [1.01–1.03]	<0.001
Causal mediation effect (c′)	0.006 [0.000–0.012]	0.047
Direct effect (c)	0.121 [0.089–0.154]	<0.001
Total effect	0.128 [0.096–0.160]	<0.001
Proportion of mediation	4.81%	
**Physical punishment**		
a	2.53 [1.85–3.21]	<0.001
b^③^	1.02 [1.01–1.03]	<0.001
Causal mediation effect (c′)	0.012 [0.005–0.019]	<0.001
Direct effect (c)	0.061 [0.027–0.095]	<0.001
Total effect	0.073 [0.040–0.107]	<0.001
Proportion of mediation	16.76%	
**Severe physical punishment**		
a	2.53 [1.85–3.21]	<0.001
b^③^	1.09 [1.07–1.12]	<0.001
Causal mediation effect (c′)	0.009 [0.005–0.012]	<0.001
Direct effect (c)	0.038 [0.024–0.052]	<0.001
Total effect	0.047 [0.033–0.061]	<0.001
Proportion of mediation	18.81%	

**Notes:**

^①^ Model adjusted for parental sex, parental age, parental ethnicity, marital status, educational achievement, residence, child sex, child age and only child status.

^②^ Coef., coefficient; OR, odds ratio; CI, confidence interval.

^③^ OR.

**Figure 2:**
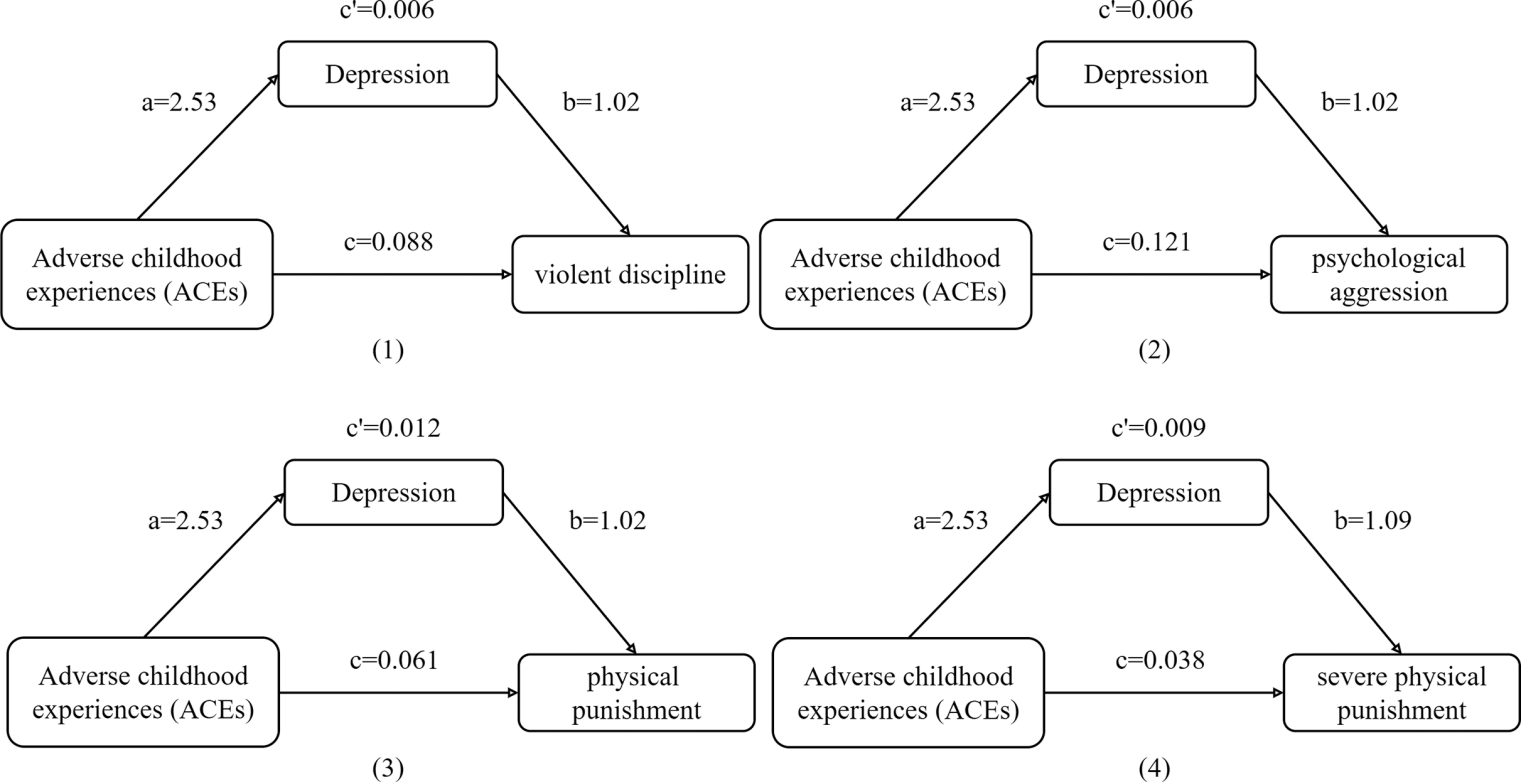
The mediating effect of depression on the relationship between ACEs and violent discipline (*n* = 1,650).

After controlling for covariates, greater parental exposure to ACEs was associated with higher depression scores (β = 2.53, *P* < 0.001) (Table S1). Furthermore, a higher depression score was associated with increased odds of violent discipline (OR 1.02, 95% CI [1.01–1.03]), psychological aggression (OR 1.02, 95% CI [1.01–1.03]), physical punishment (OR 1.02, 95% CI 1.01–1.03), and severe physical punishment (OR 1.09, 95% CI [1.07–1.12]) (Table S2). These findings suggest that higher parental exposure to ACEs is associated with greater depression severity, which in turn is associated with increased odds of violent discipline, psychological aggression, physical punishment, and severe physical punishment (Fig. 2).

## Discussion

This study examined 1,650 parents of preschool children aged 3–6 years from the Tongzhou District of Beijing. Among them, 38.4% reported experiencing at least one type of ACE, with 25.2% experiencing 1–2 types and 13.2% experiencing three or more types. Regarding parenting practices, 71.9% of parents reported using violent discipline with their children, including 63.2% engaging in psychological aggression and 48.6% using physical punishment. A significant association was observed between ACEs and violent discipline. Higher ACE exposure was associated with elevated depression scores. In turn, elevated depression scores were associated with the likelihood of violent discipline, indicating that depression acts as a mediator in the association between ACEs and violent discipline.

The prevalence of ACEs in our study was 38.4%, with our sample comprising 84.7% women and 73.5% adults aged 30–39 years. This finding is consistent with U.S. studies that used BRFSS data collected from Asian populations. For example, one study reported that 35% of Asian women aged 18–39 years experienced at least one ACE (Hurley et al., 2022). Another study of adults found a prevalence of 38.4% among Asian adults aged 30–39 years (Fisher, Stillerman & Feinglass, 2024). By contrast, our estimate is lower than the 71.4% reported among university students at the Chinese University of Hong Kong using the same BRFSS ACE questionnaire (Xu et al., 2022). This difference may be explained by variations in birth cohorts and upbringing environments, which can affect levels of ACE exposure. In addition, younger adults may be less susceptible to recall bias than older participants. The BRFSS ACE module, used in these studies as well as in our own, offers a concise alternative to longer instruments. Compared to other commonly used tools, such as the Adverse Childhood Experiences-International Questionnaire (ACE-IQ, World Health Organization, 2020), the BRFSS ACE module is much shorter. The ACE-IQ includes 29 items covering 13 ACE categories, making the BRFSS module more suitable for large-scale population surveys.

Overall, 71.9% of parents reported using violent discipline toward their children, including psychological aggression (63.2%), physical punishment (48.6%), and severe physical punishment (4.6%). These findings are consistent with previous surveys of parents of preschool children in other Chinese cities. For instance, a survey in Jiangsu found that 66.3% of mothers and 58.1% of fathers engaged in psychological aggression, while 50.1% of mothers and 45.8% of fathers used physical punishment (Cui et al., 2016). Another study in two cities in Eastern and Southern China reported prevalence rates of 69.3% for psychological aggression, 62.1% for physical punishment, and 2.2% for severe physical punishment (Qin, Du & Chen, 2023). Taken together, these results highlight the high prevalence of violent discipline among parents of preschool children in China.

The study found an association between ACEs and violent discipline, consistent with previous research in China and other Asian developing countries. For example, Zhu et al. (2024) found a significant association between ACEs and corporal punishment among parents (β = 0.59, *P* < 0.001). Similarly, Wattanatchariya, Narkpongphun & Kawilapat (2024) reported a significant association (β = 0.15, *P* < 0.01). Further supporting these findings, Shen & Wu (2024) identified a significant association between ACEs and harsh parenting, which included both corporal punishment and psychological aggression (β = 0.123, *P* < 0.05). Collectively, these studies strengthen the evidence for the link between ACEs and violent discipline in Asian parenting contexts.

We further showed that depression serves as a significant mediator between ACEs and violent discipline. To our knowledge, only one previous study within a Chinese cultural context has examined the mediating role of psychological symptoms. Using a sample from Taiwan, Shen & Wu (2024) found that psychological symptoms (including tension, depression, irritability, inferiority, and insomnia) partially mediated the relationship between parental ACEs and harsh parenting behaviors. However, this study was limited to parents of children aged 6–12 years in Taipei and New Taipei City. It did not include data from mainland China and did not specifically examine parental depression as a central mediating pathway. Our findings address this gap by demonstrating the significant mediating role of parental depression in the pathway linking ACEs to violent discipline among parents of preschool children in mainland China.

We further calculated the proportion of the total effect mediated by depression. The results were statistically significant but modest. Depression accounted for 6.55% of the total effect for violent discipline, 4.81% for psychological aggression, 16.76% for physical punishment, and 18.81% for severe physical punishment. These findings are consistent with the view that violent discipline is a multifactorial behavior. The relatively small effect size suggests that additional unmeasured pathways may contribute to this relationship. However, this should not be interpreted as a lack of practical or clinical significance. From a public health perspective, even modest effects may have meaningful implications when both the exposure and the outcome are highly prevalent. Identifying depression as a mediator is critical because it is a highly actionable factor. Unlike broad cultural shifts or socioeconomic conditions, which are challenging to modify, parental depression represents a clear and readily intervenable target. Effective interventions are well documented and include cognitive-behavioral therapy, interpersonal psychotherapy, and antidepressant medication, all of which have demonstrated clear clinical effects (National Research Council and Institute of Medicine, 2009; Boyd & Gillham, 2009). Critically, effective treatment of parental depression has been shown to directly improve parenting behaviors. For example, a meta-analysis showed that successful treatment significantly enhances parenting behaviors, such as increasing parental warmth and acceptance, improving parent-child relationship quality, and substantially reducing parenting stress (Cross et al., 2024). Another meta-analysis demonstrated that psychological treatments for perinatal depression not only alleviated maternal depressive symptoms but also improved mother-infant interaction quality, increased maternal sensitivity, and reduced parenting stress (Letourneau et al., 2017). Together, this evidence supports a clear pathway: reducing parental depression is associated with improvements in parenting behaviors, which in turn supports healthier child development outcomes. Thus, even partial mediation identifies a viable and meaningful intervention point for clinical and public health interventions aimed at mitigating the intergenerational transmission of ACEs.

This study has several strengths and limitations that should be considered when interpreting these findings. Regarding strengths, to our knowledge, this study is among the first to provide systematic evidence on the mediating role of depressive symptoms in the association between parental ACEs and violent discipline among mainland Chinese families with preschool children. This extends the literature beyond its predominant focus on Western cultural contexts. By examining this underlying psychological mechanism, our findings contribute cross-cultural evidence to the theory of intergenerational transmission of ACEs. They also highlight potentially modifiable targets for early family-based interventions tailored to the sociocultural context of mainland China. Overall, these findings may inform efforts aimed at disrupting the cycle of intergenerational violence.

However, several limitations must be acknowledged. First, as a cross-sectional study, this research is suitable for identifying associations but cannot establish causal relationships. Therefore, it remains uncertain whether effects operate in the hypothesized direction, or if alternative pathways exist. Future research should adopt a longitudinal design to establish causal relationships. Second, this study primarily relied on questionnaire surveys to obtain information, which may have introduced recall bias among respondents. Third, by focusing solely on the parental generation, the study did not assess child outcomes. Our findings illuminate one link in the intergenerational chain, from parental ACEs to parenting practices, but future research that incorporates child-level outcomes is essential to complete the picture. Finally, the generalizability of our findings is restricted by the limited geographic scope of the sample and high proportion of mothers. Participants were primarily recruited from urban and economically developed areas of Beijing. Thus, our results may not be representative of populations in rural regions or those with lower socioeconomic status in China. Additionally, mothers were overrepresented in our sample (84.7%), while fathers were underrepresented. This imbalance may limit external validity, as fathering practices and experiences may differ systematically and could not be adequately examined. Future research should expand the geographic scope, broaden the target population, and collect more representative sample data to enhance generalizability.

## Conclusion

This study found that ACEs were associated with violent discipline among Chinese parents of preschool children. Depression partially mediated the association between ACEs and violent discipline. To safeguard children’s physical and mental well-being, we recommend implementing targeted family-based intervention programs for parents who have experienced ACEs, aiming to reduce parental depression and decrease the likelihood of violent discipline toward their children.

## Supplemental Information

10.7717/peerj.21130/supp-1 Supplemental Information 1 Code.

10.7717/peerj.21130/supp-2 Supplemental Information 2 Raw data.

10.7717/peerj.21130/supp-3 Supplemental Information 3 STROBE Checklist.

10.7717/peerj.21130/supp-4 Supplemental Information 4 Association between depression and ACEs of parents (n=1650).

10.7717/peerj.21130/supp-5 Supplemental Information 5 Association between depression and violent discipline (n=1650).

## Additional Information and Declarations

### Data Availability

The following information was supplied regarding data availability:

The raw measurements and code are available in the Supplemental Files.

## References

[ref-1] Afifi TO, Salmon S, Stewart-Tufescu A, Taillieu T (2022). An examination of parents’ adverse childhood experiences (ACEs) history and reported spanking of their child: informing child maltreatment prevention efforts. International Journal of Environmental Research and Public Health.

[ref-2] Assink M, Spruit A, Schuts M, Lindauer R, van der Put CE, Stams G-JJM (2018). The intergenerational transmission of child maltreatment: a three-level meta-analysis. Child Abuse & Neglect.

[ref-3] Bhatia A, Krieger N, Victora C, Tuladhar S, Bhabha J, Beckfield J (2020). Analyzing and improving national and local child protection data in Nepal: a mixed methods study using 2014 Multiple Indicator Cluster Survey (MICS) data and interviews with 18 organizations. Child Abuse & Neglect.

[ref-4] Boyd RC, Gillham JE (2009). Review of interventions for parental depression from toddlerhood to adolescence. Current Psychiatry Reviews.

[ref-5] Bunch J, Iratzoqui A, Watts S (2018). Child abuse, self-control, and delinquency: a general strain perspective. Journal of Criminal Justice.

[ref-6] Centers for Disease Control and Prevention (2023). Behavioral risk factor surveillance system ACE data. https://www.cdc.gov/violenceprevention/aces/ace-brfss.html.

[ref-7] Choi KW, Houts R, Arseneault L, Pariante C, Sikkema KJ, Moffitt TE (2019). Maternal depression in the intergenerational transmission of childhood maltreatment and its sequelae: testing postpartum effects in a longitudinal birth cohort. Development and Psychopathology.

[ref-8] Cross M, Abdul-Karim Y, Johnson A, Victor C, Rosenfeld A (2024). Healing together: a narrative review on how psychiatric treatment for parental depression impacts children. International Journal of Environmental Research and Public Health.

[ref-9] Cui N, Xue J, Connolly CA, Liu J (2016). Does the gender of parent or child matter in child maltreatment in China?. Child Abuse & Neglect.

[ref-10] Dixon L, Browne K, Hamilton-Giachritsis C (2005). Risk factors of parents abused as children: a mediational analysis of the intergenerational continuity of child maltreatment (Part I). Journal of Child Psychology and Psychiatry, and Allied Disciplines.

[ref-11] Dube SR (2025). Reprint of: adverse childhood experiences research: the path forward. Child Abuse & Neglect.

[ref-12] Dube SR, Rishi S (2017). Utilizing the salutogenic paradigm to investigate well-being among adult survivors of childhood sexual abuse and other adversities. Child Abuse & Neglect.

[ref-13] Felitti VJ, Anda RF, Nordenberg D, Williamson DF, Spitz AM, Edwards V, Koss MP, Marks JS (1998). Relationship of childhood abuse and household dysfunction to many of the leading causes of death in adults. The Adverse Childhood Experiences (ACE) study. American Journal of Preventive Medicine.

[ref-14] Fisher C, Stillerman A, Feinglass J (2024). The association of adverse childhood experiences with household income, educational attainment and partnered status among adults aged 30–39. Child Protection and Practice.

[ref-15] Ford DC, Merrick MT, Parks SE, Breiding MJ, Gilbert LK, Edwards VJ, Dhingra SS, Barile JP, Thompson WW (2014). Examination of the factorial structure of adverse childhood experiences and recommendations for three subscale scores. Psychology of Violence.

[ref-16] Gardner MJ, Thomas HJ, Erskine HE (2019). The association between five forms of child maltreatment and depressive and anxiety disorders: a systematic review and meta-analysis. Child Abuse & Neglect.

[ref-17] Guo X, Lin L, Qin K, Li J, Chen W, Guo VY (2023). Adverse childhood experiences and depressive symptoms among middle-aged or older adults in China and the mediating role of short sleep duration. Journal of Affective Disorders.

[ref-18] Gupta S (2022). First-time exploration of adverse childhood experiences among adults in Delaware using BRFSS data: a cross-sectional study. Public Health in Practice.

[ref-19] Huang Y, Chen C, Wang Y, Gao Y, Zhou H (2020). Study on domestic violent discipline and its influencing factors for children under 5 years old in some rural areas of China. Chinese Journal of Child Health Care.

[ref-20] Hughes K, Bellis MA, Hardcastle KA, Sethi D, Butchart A, Mikton C, Jones L, Dunne MP (2017). The effect of multiple adverse childhood experiences on health: a systematic review and meta-analysis. The Lancet Public Health.

[ref-21] Hurley L, Stillerman A, Feinglass J, Percheski C (2022). Adverse childhood experiences among reproductive age women: findings from the 2019 behavioral risk factor surveillance system. Women’s Health Issues.

[ref-22] Islam MM (2024). The gradients of the relationship between child discipline practices at home and early childhood development of young children. Child Abuse & Neglect.

[ref-23] Kelley ML, Lawrence HR, Milletich RJ, Hollis BF, Henson JM (2015). Modeling risk for child abuse and harsh parenting in families with depressed and substance-abusing parents. Child Abuse & Neglect.

[ref-24] Kirkbride JB, Anglin DM, Colman I, Dykxhoorn J, Jones PB, Patalay P, Pitman A, Soneson E, Steare T, Wright T, Griffiths SL (2024). The social determinants of mental health and disorder: evidence, prevention and recommendations. World Psychiatry.

[ref-25] Lee SJ, Perron BE, Taylor CA, Guterman NB (2011). Paternal psychosocial characteristics and corporal punishment of their 3-year-old children. Journal of Interpersonal Violence.

[ref-26] Letourneau NL, Dennis C-L, Cosic N, Linder J (2017). The effect of perinatal depression treatment for mothers on parenting and child development: a systematic review. Depression and Anxiety.

[ref-27] Liu L, Wang M (2015). Parenting stress and children’s problem behavior in China: the mediating role of parental psychological aggression. Journal of Family Psychology.

[ref-28] Madigan S, Cyr C, Eirich R, Fearon RMP, Ly A, Rash C, Poole JC, Alink LRA (2019). Testing the cycle of maltreatment hypothesis: meta-analytic evidence of the intergenerational transmission of child maltreatment. Development and Psychopathology.

[ref-29] Mapp SC (2006). The effects of sexual abuse as a child on the risk of mothers physically abusing their children: a path analysis using systems theory. Child Abuse & Neglect.

[ref-30] Marçal KE (2021). Pathways to adolescent emotional and behavioral problems: an examination of maternal depression and harsh parenting. Child Abuse & Neglect.

[ref-31] McLaughlin KA, Sheridan MA, Lambert HK (2014). Childhood adversity and neural development: deprivation and threat as distinct dimensions of early experience. Neuroscience & Biobehavioral Reviews.

[ref-32] Merrick MT, Ports KA, Ford DC, Afifi TO, Gershoff ET, Grogan-Kaylor A (2017). Unpacking the impact of adverse childhood experiences on adult mental health. Child Abuse & Neglect.

[ref-33] Morelli NM, Duong J, Evans MC, Hong K, Garcia J, Ogbonnaya IN, Villodas MT (2021). Intergenerational transmission of abusive parenting: role of prospective maternal distress and family violence. Child Maltreatment.

[ref-34] Muthén B, Asparouhov T (2014). Causal effects in mediation modeling: an introduction with applications to latent variables. Structural Equation Modeling: A Multidisciplinary Journal.

[ref-35] Narayan AJ, Lieberman AF, Masten AS (2021). Intergenerational transmission and prevention of adverse childhood experiences (ACEs). Clinical Psychology Review.

[ref-36] National Research Council and Institute of Medicine (2009). Depression in parents, parenting, and children: opportunities to improve identification, treatment, and prevention.

[ref-37] Peng H, Zhang Y, Ji Y, Tang W, Li Q, Yan X, Zhuang Q (2013). Analysis of reliability and validity of Chinese version SDS scale in women of rural area. Shanghai Medical & Pharmaceutical Journal.

[ref-38] Peterson C, Aslam MV, Niolon PH, Bacon S, Bellis MA, Mercy JA, Florence C (2023). Economic burden of health conditions associated with adverse childhood experiences among US adults. JAMA Network Open.

[ref-39] Qin J, Du Y, Chen C (2023). Psychometric testing of Chinese version of ISPCAN child abuse screening tools-retrospective version: a study based on college students. Journal of Aggression, Maltreatment & Trauma.

[ref-40] Rowell T, Neal-Barnett A (2021). A systematic review of the effect of parental adverse childhood experiences on parenting and child psychopathology. Journal of Child & Adolescent Trauma.

[ref-41] Scheid CR, Miller-Graff LE, Guzmán DB (2021). Parenting practices and intergenerational cycle of victimization in Peru. Development and Psychopathology.

[ref-42] Schelbe L, Geiger JM, Schelbe L, Geiger JM (2017). Prevention and intervention strategies to address intergenerational transmission of child maltreatment. Intergenerational Transmission of Child Maltreatment.

[ref-43] Schlensog-Schuster F, Keil J, Von Klitzing K, Gniewosz G, Schulz CC, Schlesier-Michel A, Mayer S, Stadelmann S, Döhnert M, Klein AM, Sierau S, Manly JT, Sheridan MA, White LO (2024). From maltreatment to psychiatric disorders in childhood and adolescence: the relevance of emotional maltreatment. Child Maltreatment.

[ref-44] Schuetze P, Eiden RD (2005). The relationship between sexual abuse during childhood and parenting outcomes: modeling direct and indirect pathways. Child Abuse & Neglect.

[ref-45] Shen AC, Wu BC (2024). From adverse childhood experiences to harsh parenting: psychological symptoms as a mediator. Child Abuse & Neglect.

[ref-46] Shi S, Kou W, Bian Z, Chen X, Song L, Fu L, Qiu P (2025). The impact of adverse childhood experiences on cognitive function among middle-aged and older Chinese adults: multiple mediators of cognitive reserve and depressive symptoms. Journal of Affective Disorders.

[ref-47] Shin SH, Tomlinson CA, Nelson-Hence D, Ksinan Jiskrova G (2023). Understanding the intergenerational cycle of trauma and violence: maternal adverse childhood experiences and parent-to-child aggression risk. Journal of Interpersonal Violence.

[ref-48] StataCorp LLC (2024). Generalized structural equation modeling. https://www.stata.com/features/overview/generalized-sem/.

[ref-50] Swedo EA, Aslam MV, Dahlberg LL, Niolon PH, Guinn AS, Simon TR, Mercy JA (2023). Prevalence of adverse childhood experiences among U.S. adults-behavioral risk factor surveillance system, 2011–2020. MMWR. Morbidity and Mortality Weekly Report.

[ref-51] UNICEF (2014). Hidden in plain sight: a statistical analysis of violence against children. https://data.unicef.org/resources/hidden-in-plain-sight-a-statistical-analysis-of-violence-against-children/.

[ref-52] UNICEF (2017). A familiar face: violence in the lives of children and adolescents. https://data.unicef.org/resources/a-familiar-face/.

[ref-53] UNICEF (2020). Mics6 tools. https://mics.unicef.org/tools?round=53.

[ref-54] UNICEF (2024). Violent discipline. https://data.unicef.org/topic/child-protection/violence/violent-discipline/.

[ref-55] Wang W, Bian Q, Zhao Y, Li X, Wang W, Du J, Zhang G, Zhou Q, Zhao M (2014). Reliability and validity of the Chinese version of the Patient Health Questionnaire (PHQ-9) in the general population. General Hospital Psychiatry.

[ref-56] Wang M, Liu L (2014). Parental harsh discipline in mainland China: prevalence, frequency, and coexistence. Child Abuse & Neglect.

[ref-57] Wang G, Zhou Y, Duan J, Kan Q, Cheng Z, Tang S (2023). Effects of adverse childhood health experiences on cognitive function in Chinese middle-aged and older adults: mediating role of depression. BMC Public Health.

[ref-58] Wattanatchariya K, Narkpongphun A, Kawilapat S (2024). The relationship between parental adverse childhood experiences and parenting behaviors. Acta Psychologica.

[ref-59] Wei Q, Zhang C, Zhang J, Luo S, Zhao C, Wang X, Guo S (2016). Study on violent discipline and its associated factors in poor rural areas. Chinese Journal of Child Health Care.

[ref-60] Wilson S, Durbin CE (2010). Effects of paternal depression on fathers’ parenting behaviors: a meta-analytic review. Clinical Psychology Review.

[ref-61] Wolford SN, Cooper AN, McWey LM (2019). Maternal depression, maltreatment history, and child outcomes: the role of harsh parenting. The American Journal of Orthopsychiatry.

[ref-62] World Health Organization (2020). Adverse Childhood Experiences International Questionnaire (ACE-IQ). https://www.who.int/publications/m/item/adverse-childhood-experiences-international-questionnaire-(ace-iq).

[ref-63] Xie Y, Fang Y, Zeng Y (2023). The incidence of adverse childhood experiences among residents over 45 years old and health effects in China. Chinese Journal of Health Statistics.

[ref-64] Xu Z, Zhang D, Ding H, Zheng X, Lee RC, Yang Z, Mo PK, Lee EK, Wong SY (2022). Association of positive and adverse childhood experiences with risky behaviours and mental health indicators among Chinese university students in Hong Kong: an exploratory study. European Journal of Psychotraumatology.

[ref-65] Yu P, Wang X, Liu J, Luo H, Yi Y (2024). Adverse childhood experiences, marital status and depressive symptoms in later life among the Chinese middle-aged and older adults: the mediating role of marital status. BMC Public Health.

[ref-66] Zhang T, Kan L, Jin C, Shi W (2023). Adverse childhood experiences and their impacts on subsequent depression and cognitive impairment in Chinese adults: a nationwide multi-center study. Journal of Affective Disorders.

[ref-67] Zhang D, Luo J, Peng L, Yu Z, Li L, Sun R, Wu X (2012). Factor analysis on survey results of the self rating depression scale (SDS) in students. Journal of Kunming Medical University.

[ref-68] Zhu J, Racine N, Devereux C, Hodgins DC, Madigan S (2025). Associations between adverse childhood experiences and substance use: a meta-analysis. Child Abuse & Neglect.

[ref-69] Zhu Y, Zhang G, Zhan S, Anme T (2024). Longitudinal effects of parental adverse childhood experiences on offspring problematic media use: the serial mediating role of psychological distress and harsh discipline. Child Abuse & Neglect.

[ref-70] Zung WW, Richards CB, Short MJ (1965). Self-rating depression scale in an outpatient clinic. Further validation of the SDS. Archives of General Psychiatry.

