# Supplementary material for: The mediating role of depression between adverse childhood experiences and violent discipline among Chinese parents of preschool children
Source: PeerJ. 2026 Apr 28;14:e21130. doi: 10.7717/peerj.21130 (PMC13134545; doi:10.7717/peerj.21130)
Supplement: Supplemental Information 4 [file peerj-14-21130-s004.docx]

**Supplementary Table 1 Association between depression and ACEs of parents (n=1650)**

|  | **Depression** |
| --- | --- |
|  | **Coef. (95% CI)^①②^** |
| **ACEs^③^** | 2.53^***^ (1.85,3.21) |
| **Parental sex** |  |
| (Male) |  |
| Female | -0.55 (-1.91,0.81) |
| **Parental age** |  |
| (>=40) |  |
| <30 | 0.77 (-1.28,2.81) |
| >=30 & <35 | -0.37(-1.83,1.10) |
| >=35 & <40 | 0.77 (-0.69,2.24) |
| **Parental Ethnicity** |  |
| (Han) |  |
| Minority | -2.09^*^ (-4.12,-0.05) |
| **Marital status** |  |
| (married) |  |
| otherwise | 1.79 (-2.19,5.77) |
| **Educational achievement** |  |
| (Master’s or above) |  |
| Middle school or below | 6.16^***^ (3.23,9.10) |
| High school or vocational school | 5.06^***^ (2.83,7.30) |
| College or university | 1.57 (-0.12,3.26) |
| **Residence** |  |
| (urban) |  |
| rural | 2.05^**^ (0.52,3.59) |
| **Child sex** |  |
| (Male) |  |
| Female | 0.53 (-0.45,1.50) |
| **Child age** |  |
| (3 years old) |  |
| 4 years old | 0.05 (-1.21,1.31) |
| 5-6 years old | -0.30 (-1.56,0.96) |
| **Only child** |  |
| (Yes) |  |
| No | -0.42 (-1.45,0.60) |

^①^ * p < 0.05, ** p < 0.01, *** p < 0.001.

^②^ CI, confidence interval

^③^ To facilitate the presentation in Table 4 and Figure 2, ACEs are treated as a continuous variable, indicating that for each increase in cumulative exposure to ACEs by one level, the average expected change in depression scores occurs.
