# Supplementary material for: The mediating role of depression between adverse childhood experiences and violent discipline among Chinese parents of preschool children
Source: PeerJ. 2026 Apr 28;14:e21130. doi: 10.7717/peerj.21130 (PMC13134545; doi:10.7717/peerj.21130)
Supplement: Supplemental Information 5 [file peerj-14-21130-s005.docx]

**Supplementary Table 2 Association between depression and violent discipline (n=1650)**

| **Characteristic** | **Violent discipline** | **Psychological aggression** | **Physical punishment** | **Severe physical punishment** |
| --- | --- | --- | --- | --- |
|  | **OR (95% CI)^①②^** | **OR (95% CI)^①②^** | **OR (95% CI)^①②^** | **OR (95% CI)^①②^** |
| **Depression** | 1.02^**^ (1.01,1.03) | 1.02^***^ (1.01,1.03) | 1.02^***^ (1.01,1.03) | 1.09^***^ (1.07,1.12) |
| **Parental sex** |  |  |  |  |
| (Male) | 1.00 | 1.00 | 1.00 | 1.00 |
| Female | 0.92 (0.67,1.25) | 0.97 (0.73,1.29) | 0.84 (0.64,1.11) | 0.74 (0.40,1.37) |
| **Parental age** |  |  |  |  |
| (>=40) | 1.00 | 1.00 | 1.00 | 1.00 |
| <30 | 1.55 (0.98,2.44) | 1.78^**^ (1.16,2.73) | 1.15 (0.76,1.74) | 1.91 (0.77,4.72) |
| >=30 & <35 | 1.49^*^ (1.08,2.05) | 1.70^***^ (1.25,2.29) | 1.21 (0.90,1.62) | 1.35 (0.65,2.79) |
| >=35 & <40 | 1.39^*^ (1.01,1.91) | 1.51^**^ (1.12,2.04) | 1.07 (0.79,1.44) | 0.79 (0.37,1.71) |
| **Parental Ethnicity** |  |  |  |  |
| (Han) | 1.00 | 1.00 | 1.00 | 1.00 |
| Minority | 1.34 (0.83,2.17) | 0.91 (0.59,1.38) | 1.37 (0.91,2.07) | 1.11 (0.38,3.24) |
| **Marital status** |  |  |  |  |
| (married) | 1.00 | 1.00 | 1.00 | 1.00 |
| otherwise | 1.15 (0.45,2.95) | 1.22 (0.52,2.91) | 1.61 (0.71,3.67) | 1.89 (0.40,8.83) |
| **Educational achievement** |  |  |  |  |
| (Master's or above) | 1.00 | 1.00 | 1.00 | 1.00 |
| Middle school or below | 0.75 (0.40,1.42) | 0.56 (0.31,1.03) | 1.48 (0.81,2.71) | 1.22 (0.31,4.84) |
| High school or vocational school | 1.21 (0.73,2.01) | 1.09 (0.68,1.75) | 1.71^*^ (1.08,2.72) | 1.39 (0.46,4.26) |
| College or university | 1.13 (0.78,1.64) | 1.11 (0.79,1.58) | 1.45^*^ (1.03,2.06) | 0.97 (0.37,2.55) |
| **Residence** |  |  |  |  |
| (urban) | 1.00 | 1.00 | 1.00 | 1.00 |
| rural | 0.97 (0.68,1.38) | 0.92 (0.66,1.27) | 0.96 (0.70,1.31) | 0.61 (0.28,1.29) |
| **Child sex** |  |  |  |  |
| (Male) | 1.00 | 1.00 | 1.00 | 1.00 |
| Female | 0.88 (0.70,1.09) | 0.97 (0.79,1.19) | 0.73^**^ (0.60,0.89) | 0.40^***^ (0.24,0.67) |
| **Child age** |  |  |  |  |
| (3 years old) | 1.00 | 1.00 | 1.00 | 1.00 |
| 4 years old | 1.37^*^ (1.04,1.81) | 1.50^**^ (1.15,1.95) | 1.18 (0.92,1.53) | 1.68 (0.89,3.16) |
| 5-6 years old | 1.44^*^ (1.09,1.90) | 1.44^**^ (1.11,1.86) | 1.00 (0.78,1.30) | 1.10 (0.55,2.18) |
| **Only child** |  |  |  |  |
| (Yes) | 1.00 | 1.00 | 1.00 | 1.00 |
| No | 1.07 (0.85,1.35) | 1.05 (0.85,1.30) | 1.24^*^ (1.00,1.52) | 1.39 (0.84,2.30) |

^①^ * p < 0.05, ** p < 0.01, *** p < 0.001.

^②^ OR, odds ratio; CI, confidence interval
